# Emerging Role of Oral Mesenchymal Stem/Stromal Cells and Their Derivates

**DOI:** 10.3390/ijms241512003

**Published:** 2023-07-26

**Authors:** Guya Diletta Marconi, Francesca Diomede, Jacopo Pizzicannella, Oriana Trubiani

**Affiliations:** 1 Department of Innovative Technologies in Medicine & Dentistry, University “G. d’Annunzio” Chieti-Pescara, Via dei Vestini, 31, 66100 Chieti, Italy; guya.marconi@unich.it (G.D.M.); francesca.diomede@unich.it (F.D.); 2 Department of Engineering and Geology, University “G. d’ Annunzio” Chieti-Pescara, Viale Pindaro, 42, 65127 Pescara, Italy; jacopo.pizzicannella@unich.it

## 1. Introduction

Mesenchymal stem/stromal cells (MSCs) have fewer ethical, moral, and safety problems in comparison with embryonic stem cells [1]. MSCs were firstly discovered in the bone marrow (BM-MSCs), but they can be isolated from other sources, including tissues and organs, among others, such as the lungs, muscles, adipose tissue, placenta, umbilical cord, dermis, and dental tissue [2].

**Figure 1:**
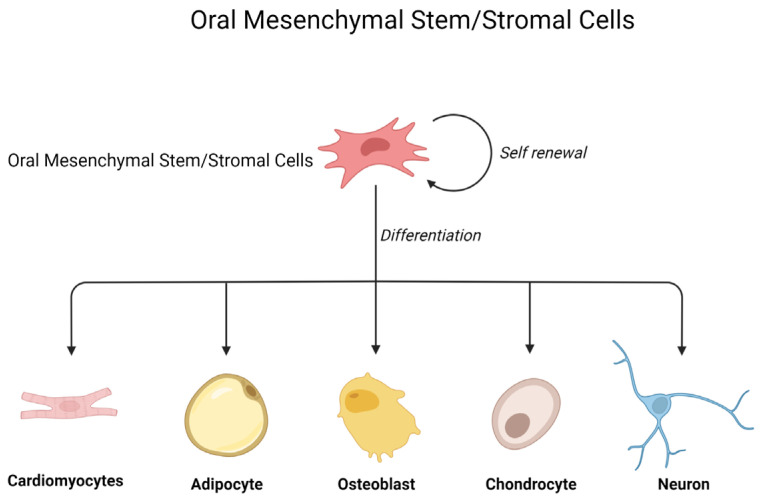
Oral mesenchymal stem/stromal cell differentiation (created with BioRender.com).

MSCs are characterized by the expression of specific surface molecules (such as CD90, STRO-1, CD105, and CD73), adherence to plastic in culture, and the capability of differentiating into chondrocytes, adipocytes, osteocytes, cardiomyocytes, and neurocytes [3] (Figure 1).

Human MSCs isolated from oral tissues possess long-term proliferation ability and multipotency properties that are exploited for clinical purposes, including tissue regeneration and immunomodulation [4,5]. MSCs can mediate paracrine action by secreting MSC-EVs [6,7].

Extracellular vesicles (EVs) are secreted by different cell types, and those produced by oral-cavity-derived mesenchymal stem/stromal cells (OMSCs), including human gingival mesenchymal stem cells (hGMSCs), have proangiogenic and anti-inflammatory effects, showing a potentially therapeutic role in tissue regeneration [8].

Moreover, the latest in vitro and in vivo studies on hOMSCs exhibited their capability to produce not only a large quantity of cytokines but also EVs with high contents of anti-inflammatory mediators, resulting in them being important for therapeutic strategies for several diseases, in addition to the regenerative capacity of damaged tissues [9].

The applications and mechanisms of EVs are gaining a lot of interest in the current scientific research, as EVs may take part in several instances of intercellular communication in different tissues. Based on their immunoregulatory function and regenerative ability, OMSC-EVs can be extensively used as specific biological macromolecules in the paracrine signaling pathway [10].

## 2. Extracellular Vesicles Derived from Oral Mesenchymal Stem Cells and Their Regenerative and Immunomodulation Potential

**Figure 2:**
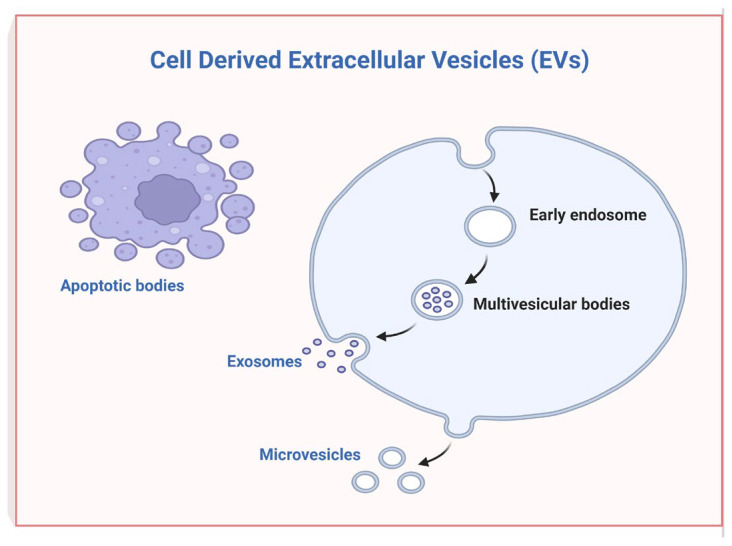
Cell-derived extracellular vesicles (EVs) (created with BioRender.com).

Human MSCs from dental tissues, dental pulp stem cells (DPSCs), stem cells from the apical papilla (SCAPs), periodontal ligament stem cells (PDLSCs), gingival-derived MSCs (GMSCs), dental follicle stem cells (DFSCs), tooth germ stem cells (TGSCs), and alveolar-bone-derived MSCs (ABMSCs) were isolated [11]. The paracrine features of MSCs are operated through secreting soluble factors and liberating EVs, such as exosomes and microvesicles [12]. EVs are mostly endosomal in origin and enclose a cargo of miRNA, mRNA, and proteins that are transferred from their original cells to target cells. It has recently emerged that EVs alone are responsible for the therapeutic effect of MSCs. In detail, EVs are lipid-bilayer-bound vesicles released by cells with the characteristic of being implicated in intercellular communication [13]. Released membrane vesicles from eukaryotic cells, such as exosomes, microparticles, microvesicles, and apoptotic bodies, can be retained as a dynamic extracellular vesicular compartment, strategic for their paracrine or autocrine biological effects on tissue metabolism (Figure 2).

Due to the low immunogenicity, elevated safety, and efficiency of MSC-EVs, MSC-EVs may serve as novel therapeutic agents for tissue engineering and regenerative medicine. OMSCs release EVs of varied miRNA profiles to induce osteogenic differentiation and extracellular matrix mineralization [14]. The cytokines and miRNAs encapsulated in MSC-EVs may accelerate the process of fracture healing. Certain miRNAs, such as miR-21, miR-4532, miR-125b-5p, and miR-338-3p, may play a regulatory role in bone formation and angiogenesis [15]. In particular, the secretome from hOMSCs represents a possible candidate for a novel cell-free therapy overcoming the limitations and risks of cell-based therapies, including immune incompetency, carcinogenicity, conditions for ex vivo cell expansion, and costs [16].

In our previous studies, it was reported that treatment with a conditioned medium derived from hPDLSCs under hypoxia (H-hPDLSCs-CM) strongly inhibits experimental autoimmune encephalomyelitis (EAE) and clinical impact, mainly reducing the inflammatory pathway [17]. EVs represent intercellular communication systems able to connect with target cells by binding to cell surface receptors, transferring membrane proteins, and merging their membrane contents into recipient-cell plasma membranes [18].

For these reasons, the application of hPDLSC-derived EVs may provide a novel potential tool for tissue engineering and regenerative medicine [19].

## 3. Conclusions

In conclusion, stem-cell-free therapy and, in particular, the released secretome from hOMSCs could be take into consideration as an alternative and promising therapeutic tool.

## Data Availability

Data are available to the corresponding author upon request.
